# Protective Effector Memory CD4 T Cells Depend on ICOS for Survival

**DOI:** 10.1371/journal.pone.0016529

**Published:** 2011-02-18

**Authors:** Tamson V. Moore, Bryan S. Clay, Caroline M. Ferreira, Jesse W. Williams, Magdalena Rogozinska, Judy L. Cannon, Rebecca A. Shilling, Amanda L. Marzo, Anne I. Sperling

**Affiliations:** 1 Committee on Immunology & Section of Pulmonary and Critical Care Medicine, Department of Medicine, The University of Chicago, Chicago, Illinois, United States of America; 2 Department of Immunology and Microbiology, Rush University Medical Center, Chicago, Illinois, United States of America; Centre de Recherche Public de la Santé (CRP-Santé), Luxembourg

## Abstract

Memory CD4 T cells play a vital role in protection against re-infection by pathogens as diverse as helminthes or influenza viruses. Inducible costimulator (ICOS) is highly expressed on memory CD4 T cells and has been shown to augment proliferation and survival of activated CD4 T cells. However, the role of ICOS costimulation on the development and maintenance of memory CD4 T cells remains controversial. Herein, we describe a significant defect in the number of effector memory (EM) phenotype cells in ICOS^−/−^ and ICOSL^−/−^ mice that becomes progressively more dramatic as the mice age. This decrease was not due to a defect in the homeostatic proliferation of EM phenotype CD4 T cells in ICOS^−/−^ or ICOSL^−/−^ mice. To determine whether ICOS regulated the development or survival of EM CD4 T cells, we utilized an adoptive transfer model. We found no defect in development of EM CD4 T cells, but long-term survival of ICOS^−/−^ EM CD4 T cells was significantly compromised compared to wild-type cells. The defect in survival was specific to EM cells as the central memory (CM) ICOS^−/−^ CD4 T cells persisted as well as wild type cells. To determine the physiological consequences of a specific defect in EM CD4 T cells, wild-type and ICOS^−/−^ mice were infected with influenza virus. ICOS^−/−^ mice developed significantly fewer influenza-specific EM CD4 T cells and were more susceptible to re-infection than wild-type mice. Collectively, our findings demonstrate a role for ICOS costimulation in the maintenance of EM but not CM CD4 T cells.

## Introduction

Memory CD4 T cells are a critical component of protective immunity to disease [1], [2], [3], [4], [5], [6], [7] as well as many pathological immune responses [8], [9], [10], [11], [12]. Two classes of memory CD4 T cells with unique biological roles have been distinguished by differential expression of lymphoid tissue-homing molecules: lymphoid-homing central memory cells (CD44^hi^CD62L^+^CCR7^+^) and circulating and tissue-homing effector memory cells (CD44^hi^CD62L^−^CCR7^−^) [13]. Effector memory (EM) CD4 T cells have been shown to be highly differentiated and produce effector cytokines (such as IL-4 or IFN-γ) more rapidly than central memory (CM) CD4 cells [13], [14], [15]. Conversely, CM CD4 cells have greater proliferative capacity and may be able to differentiate to multiple lineages after re-activation [16], [17]. The survival and homeostatic proliferation of CM and EM CD4 T cells are regulated differently: CM CD4 T cells express higher levels of anti-apoptotic signaling molecules whereas EM CD4 T cells undergo greater levels of homeostatic proliferation [18], [19]. Collectively, these findings suggest that central and effector memory CD4 T cells occupy distinct niches.

Costimulatory molecules that enhance proliferation and survival (CD28 and OX40) have been found to enhance the development of memory CD4 T cells [20], [21], [22]. As EM CD4 T cells are thought to derive from highly proliferated CD4 T cells and ICOS costimulation has been found to enhance proliferation of CD4 T cells [23], we hypothesized that ICOS costimulation might play a role in the development or homeostatic proliferation of EM CD4 T cells.

In this study, we find that in the absence of ICOS costimulation, there is reduced survival of EM but not CM CD4 T cells. The reduced population of EM CD4 T cells is associated with a reduced population of cytokine-producing cells and reduced protection against re-infection in ICOS^−/−^ mice. Collectively, our results demonstrate that ICOS costimulation regulates the survival of protective effector memory CD4 T cells.

## Results

### ICOS-deficient mice have fewer effector memory phenotype CD4 T cells

**Figure 1:**
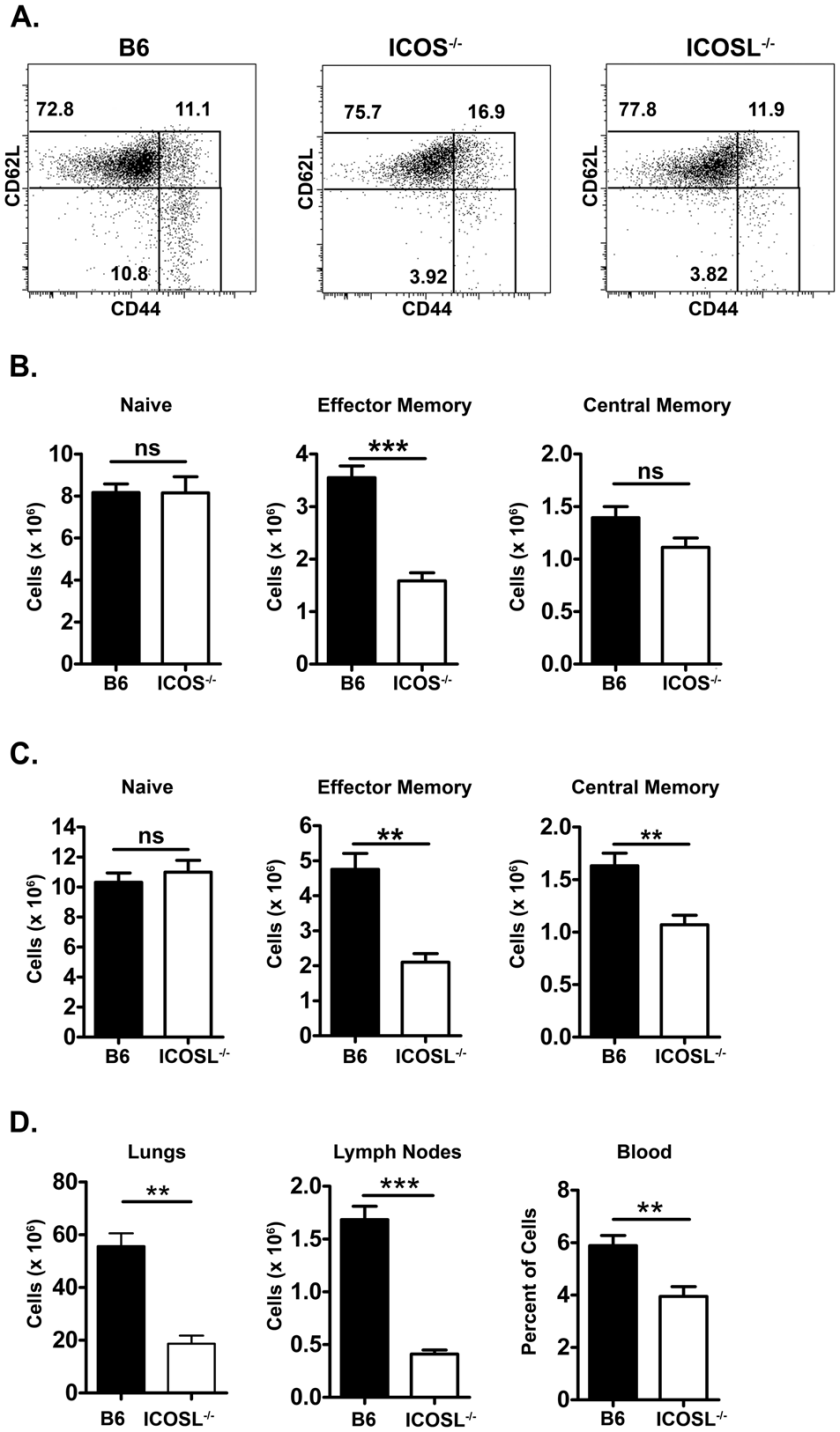
ICOS^−/−^ and ICOSL^−/−^ mice have fewer effector memory phenotype CD4 T cells. Age-matched wild-type and ICOS^−/−^ (B) or ICOSL^−/−^ (C & D) mice were sacrificed. Splenocytes (A–C) or cells from the lungs, lymph nodes, and blood (D) were harvested and stained for CD3, CD4, CD44 and CD62L. Cells were gated on CD3^+^CD4^+^ cells then on CD44 and CD62L. Naïve cells are identified as CD44^−^CD62L^+^, effector memory cells as CD44^+^CD62L^−^, and central memory cells as CD44^+^CD62L^+^. Representative plots with gating on naïve, central memory and effector memory populations in wild-type, ICOS^−/−^, and ICOSL^−/−^ mice are shown (A). The total numbers of naïve, effector memory phenotype and central memory phenotype cells in the organs of wild-type and ICOS^−/−^ or ICOSL^−/−^ mice were calculated from the percent of each population and total numbers of cells in the organ. N≥4 for each group.

To determine whether ICOS might regulate the development or survival of memory CD4 T cells, we investigated the pre-existing population of memory phenotype CD4 T cells in untreated ICOS^−/−^ and ICOSL^−/−^ mice compared to wild-type mice. Central memory phenotype CD4 T cells were identified as CD44^high^CD62L^high^ CD4 T cells and EM phenotype CD4 T cells were gated on CD44^high^CD62L^low^ CD4 T cells (Figure 1A), as previously described [13], [24]. ICOS^−/−^ mice had similar numbers of naïve and central memory phenotype CD4 T cells compared to wild-type mice but had a significant defect in the number of EM phenotype CD4 T cells (Figure 1B). Similar to ICOS^−/−^ mice, ICOSL^−/−^ mice had no defect in naïve cell numbers and a dramatic defect in EM phenotype CD4 T cell numbers (Figure 1C). ICOSL^−/−^ mice also had a significant defect in the number of central memory phenotype CD4 T cells, but this was neither as consistent nor as dramatic as the defect observed in the EM phenotype CD4 T cell population.

As well as being found in the spleen, EM CD4 T cells are also found in the tissues, and a small population of EM CD4 T cells traffics through the lymph nodes. Moreover, the spleen includes both circulating cells and lymphoid-homing cells, and therefore does not completely represent circulating cells. We investigated the number of EM phenotype CD4 T cells in the blood, the lymph nodes, and the lungs, which we chose as a representative tissue that is highly exposed to environmental antigens. We found that ICOS^−/−^ and ICOSL^−/−^ mice had fewer EM phenotype CD4 T cells in all tissues examined (Figure 1D). Conversely, there was no difference in central memory CD4 T cells and while there sometimes appeared to be a defect in the CM and EM CD8 T cells in ICOS^−/−^ and ICOSL^−/−^ mice, it was neither as consistent nor as dramatic as the defect in ICOS^−/−^ EM CD4 T cells (data not shown). Collectively, these results indicate that ICOS costimulation regulates either the development or maintenance of EM phenotype CD4 T cells that are present in the lymphoid and non-lymphoid tissues.

**Figure 2:**
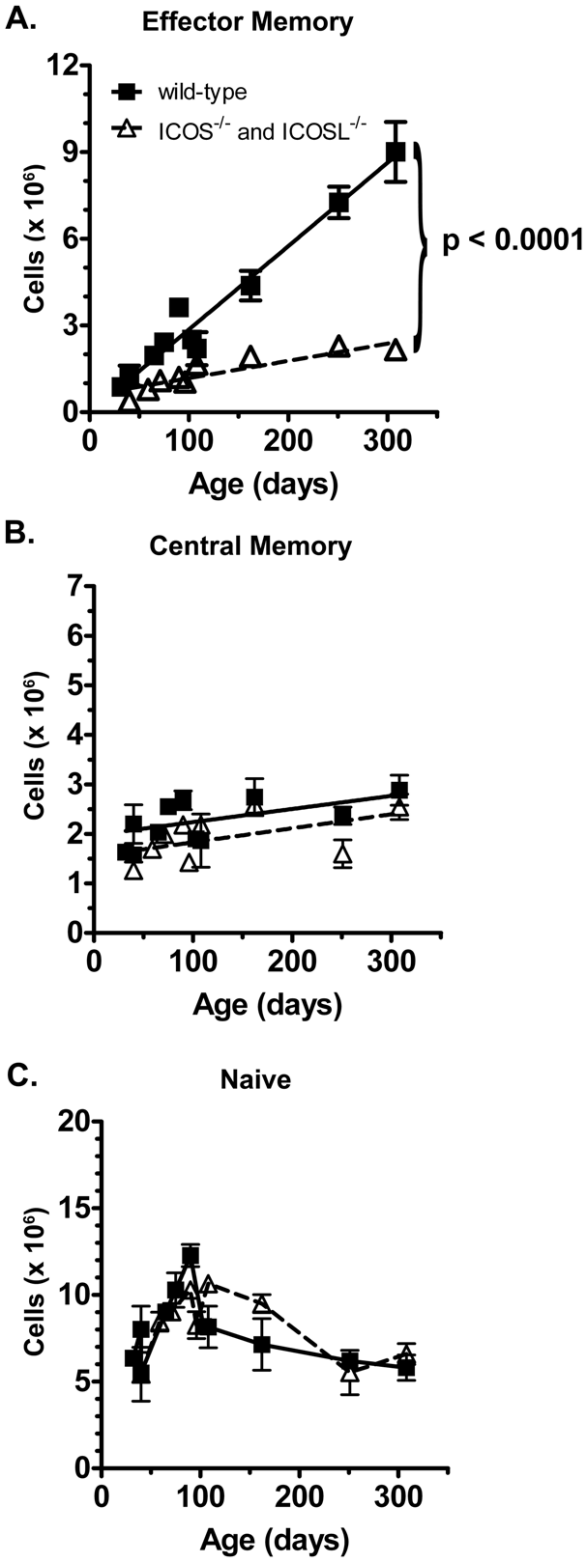
Effector memory CD4 T cells accumulate at a significantly reduced rate in ICOS-deficient mice. Wild-type and ICOS^−/−^ or ICOSL^−/−^ (ICOS-deficient) mice of various ages were sacrificed. Splenocytes were harvested and stained for flow cytometry as described in Figure 1. The total number of naïve, effector memory phenotype and central memory phenotype cells in the spleens of wild-type and ICOS-deficient mice of each age were calculated. For the effector memory phenotype cells, linear regression was performed and the p-value for effector memory phenotype cells (A) represents the likelihood of the slopes of the linear regressions being identical. 2-way ANOVA analysis of the data where there were precisely age-matched mice revealed similar significance.

EM phenotype CD4 T cells have been shown to accumulate with age in untreated mice and humans [25], [26], [27]. We investigated whether there was a difference in this accumulation of memory cells with age in untreated wild-type (B6) and ICOS-deficient (B6 ICOS^−/−^ and ICOSL^−/−^) mice. Strikingly, the rate of accumulation of EM phenotype CD4 cells was approximately five-fold less in the absence of ICOS costimulation (Figure 2A). In contrast, there was no defect in the populations of naïve or central memory phenotype cells in ICOS-deficient mice (Figure 2B). Nor did ICOS-deficient mice have a reduced population of naïve CD4 T cells at any age (Figure 2C). These results suggest that ICOS costimulation enhances either the development or maintenance of effector memory phenotype cells.

### ICOS-deficient effector memory phenotype CD4 T cells have no defect in homeostatic proliferation

**Figure 3:**
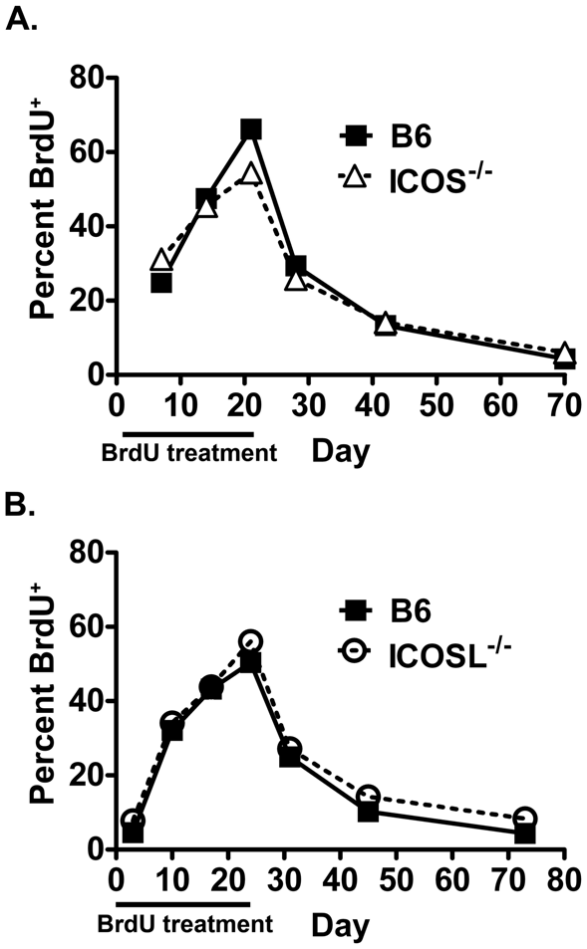
ICOS-deficient and wild-type effector memory phenotype CD4 T cells undergo similar homeostatic proliferation. A. Wild-type and ICOS^−/−^ mice were treated with BrdU for 21 days, then followed for a further 49 days without BrdU. Blood was drawn periodically and red blood cell-depleted. The lymphocytes were then stained for CD4, CD44, CD62L, and BrdU and analyzed by flow cytometry. The percent BrdU^+^ of CD44^+^CD62L^−^ CD4^+^ cells was assessed at each time point. B. Wild-type and ICOSL^−/−^ mice were treated with BrdU for 24 days, followed for 49 days, and analyzed similarly to A. N≥4 for all groups.

The defect in EM phenotype cells in ICOS^−/−^ mice was dramatic and suggested that there might either be a defect in development or reduced homeostatic proliferation of these cells. As EM phenotype cells develop in response to unknown antigens, there was no straightforward way to test the development of the cells. However, the homeostatic proliferation of these cells could be tracked by treating mice with the thymidine analogue BrdU, which is incorporated into the DNA of proliferating cells and detectable by intracellular staining and flow cytometry. In two experiments, wild-type and ICOS^−/−^ or wild-type and ICOSL^−/−^ mice were treated with BrdU, and BrdU incorporation during treatment (due to proliferation) and BrdU loss after treatment (due to proliferation and to death) were assessed. EM CD4 T cells in ICOS^−/−^ (Figure 3A) and ICOSL^−/−^ mice (Figure 3B) incorporated BrdU at similar rates during treatment as EM CD4 T cells in wild-type mice. Similarly, after stopping treatment, EM CD4 T cells lost BrdU at similar rates in ICOS^−/−^ and ICOSL^−/−^ mice as in wild-type mice. These findings indicate that the dramatic reduction in EM phenotype CD4 T cells in ICOS^−/−^ and ICOSL^−/−^ mice is not attributable to differences in homeostatic proliferation.

### ICOS deficiency reduces antigen-specific effector memory CD4 T cell survival

**Figure 4:**
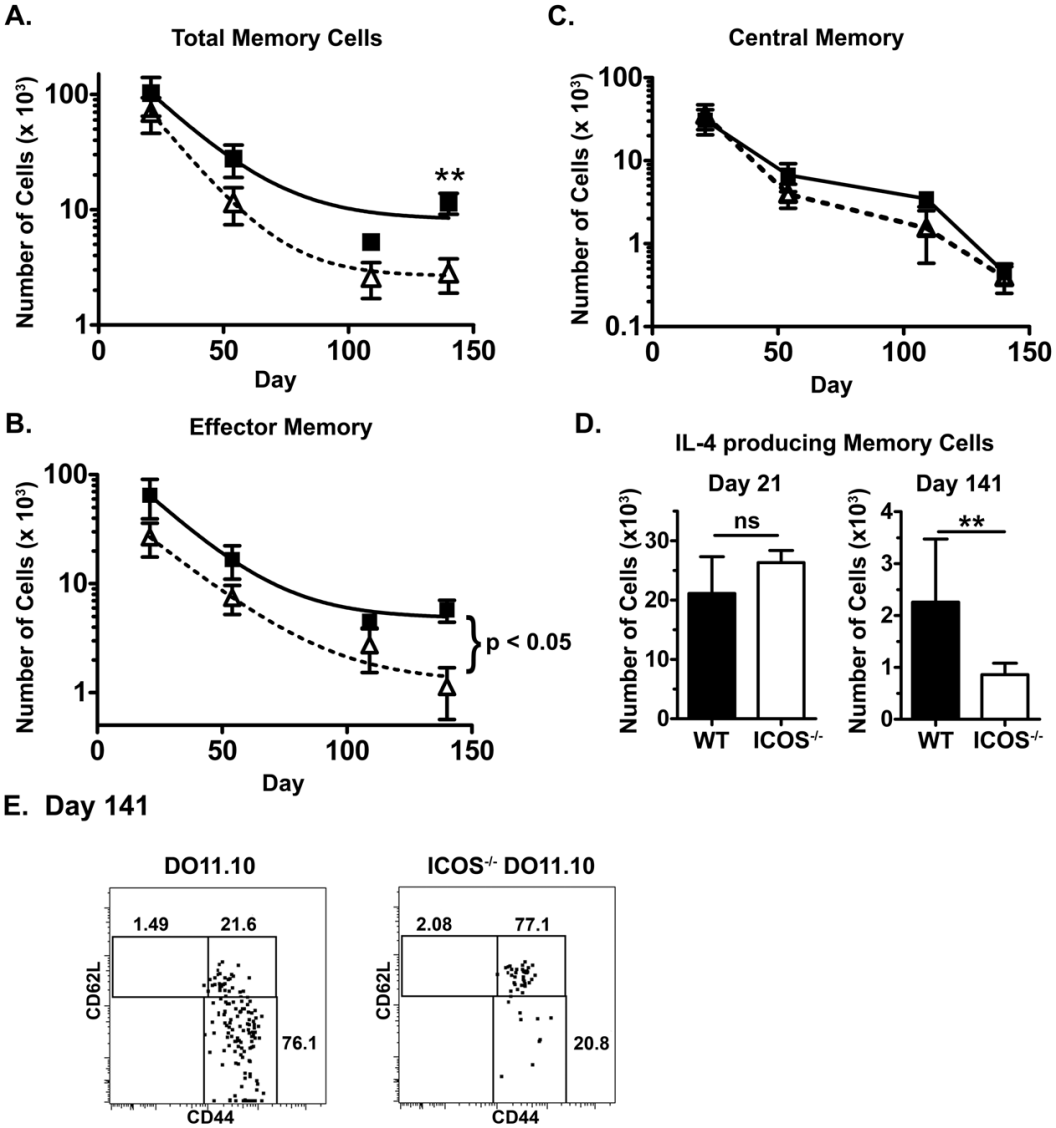
ICOS^−/−^ effector memory but not central memory CD4 T cells have a defect in survival. Wild-type (filled squares, solid black line) and ICOS^−/−^ (open triangles, dotted lines) DO11.10 cells were adoptively transferred into separate hosts that were immunized the next day with OVAp and inactivated S. mansonii eggs, as previously described. On various days after immunization, hosts were sacrificed and the spleen and lymph nodes were harvested. Spleen and lymph node cells were stained with biotin-labeled KJ1-26 antibody, enriched with anti-biotin MACS beads, and then stained further for CD4, CD44, and CD62L and with a streptavidin-fluorochrome reagent to bind biotinylated KJ1-26 antibody. At some time-points, a fraction of the isolated cells were restimulated for 48 hours with OVAp to identify cytokine-producing cells (D). A. Total numbers of CD4^+^KJ1-26^+^ cells. B. Number of effector memory (CD44^+^CD62L^low^) CD4^+^KJ1-26^+^ cells. C. Number of central memory (CD44^+^CD62L^high^) CD4^+^KJ1-26^+^ cells. Significance (p-value) in part B. reflects the likelihood that the curves are the same (i.e. that one curve would represent all of the data as well as two separate curves for wild-type and ICOS^−/−^ cells). D. Total numbers of IL-4-producing KJ1-26-enriched cells on day 140. E. Sample plots of DO11.10 and ICOS^−/−^ DO11.0 memory populations at day 140. N≥4 for every group at each time-point.

As the reduced number of EM CD4 T cells in ICOS^−/−^ mice could not be attributed to proliferation, we hypothesized that this deficiency could be either due to a defect in generation or in survival of EM CD4 T cells. To follow the generation and survival of wild-type and ICOS^−/−^ EM CD4 T cells, we utilized an adoptive transfer model. Naïve (CD62L^high^) wild-type or ICOS^−/−^ DO11.10 cells were transferred into hosts and the hosts were subsequently immunized. Memory cell populations were followed for over four months after the contraction of the primary response. We found that the ICOS^−/−^ memory CD4 T cell population declined at a greater rate than the wild-type population (Figure 4A), which was attributable to decreases in the EM CD4 T cell population (Figure 4B) while there was little or no difference in the CM CD4 T cell population (Figure 4C). Interestingly, at early time points (days 21 & 54), there was not a significant difference in the number of ICOS^−/−^ and wild-type EM CD4 T cells (Figure 4B), suggesting that the development EM CD4 T cells was not significantly impaired by the absence of ICOS costimulation. However, by day 141, the ICOS^−/−^ CD4 memory cells were largely CD62L^high^ central memory cells while there was a significant population of wild-type CD62L^low^ EM cells (Cell counts in Figures 4A–C, representative graphs shown in Figure 4E). From these data, we concluded that ICOS costimulation enhanced antigen-specific effector memory cell persistence.

### ICOS costimulation enhances the long-term persistence of IL-4-producing effector memory CD4 T cells

To determine whether the reduced numbers of ICOS^−/−^ memory CD4 T cells was associated with a reduction in cytokine-producing memory CD4 T cells, memory CD4 T cells (enriched for TCR-transgenic cells) were restimulated in an ELISPOT assay. We quantified the numbers of IL-4-producing ICOS^−/−^ and wild-type TCR-transgenic cells at the early (day 21) and late (day 140) memory time points. There were similar numbers of ICOS^−/−^ and wild-type total memory cells (Figure 4A) and IL-4-producing memory cells (Figure 4D) early after the contraction of the immune response. After the cells had rested an additional four months, there were severe defects in both the number of ICOS^−/−^ effector memory cells (Figure 4B) and the number of IL-4-producing memory cells (Figure 4D, right). Collectively, these data suggest that ICOS costimulation enhances the long-term persistence of IL-4-producing EM CD4 T cells.

### ICOS-deficient mice are more susceptible to influenza, and develop fewer effector memory CD4 T cells in response to influenza infection

To determine the role of ICOS costimulation on the development of memory in a physiological response, wild-type and ICOS^−/−^ or ICOSL^−/−^ mice were infected with the PR8 strain of influenza A, and antigen-specific CD4 T cells were identified by staining and enriching memory cells with a hemagglutinin (HA, epitope 143–155)-specific I-A^d^ tetramer (HA-tetramer), as previously described by Moon et al. [28], [29].

**Figure 5:**
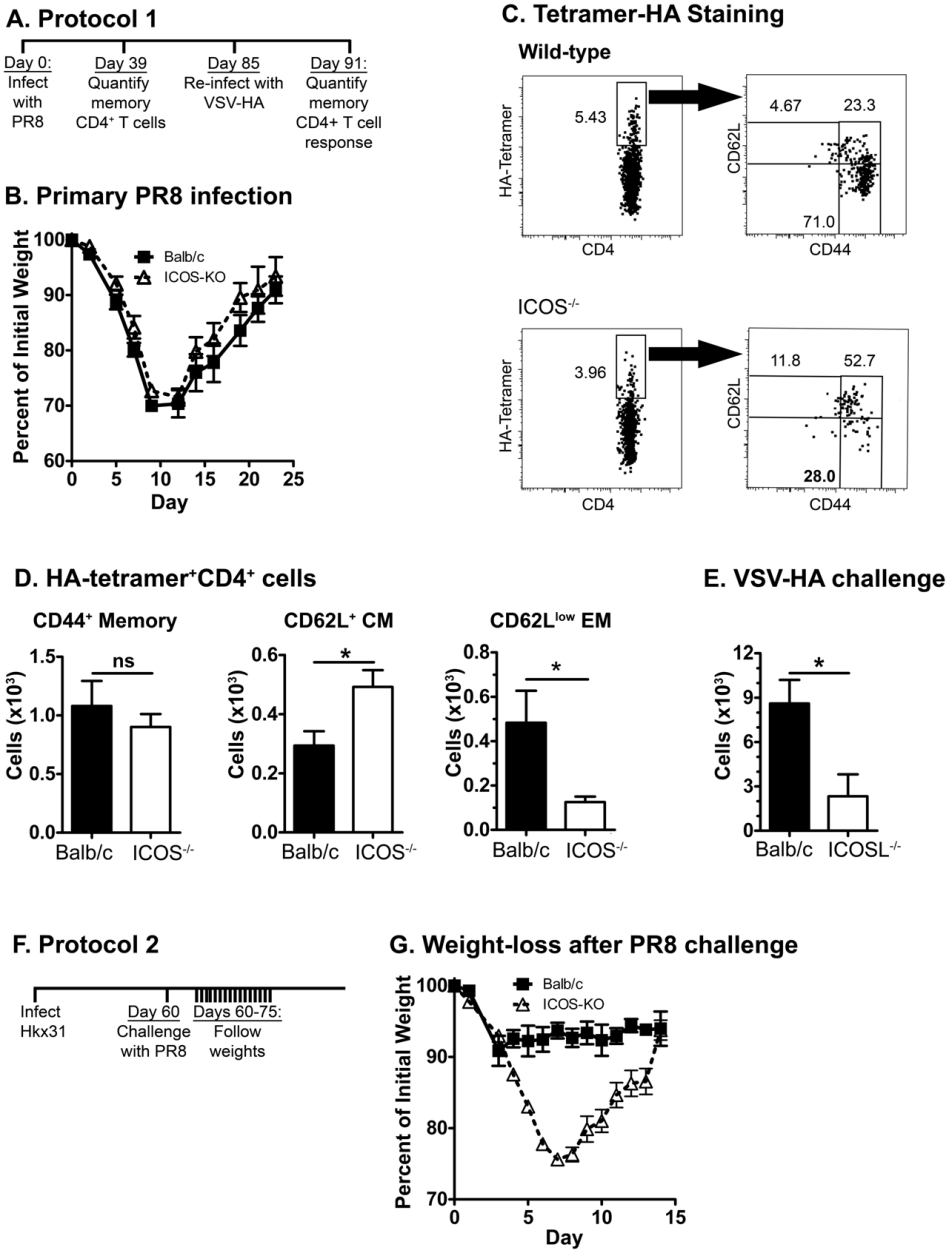
ICOS^−/−^ mice develop fewer effector memory cells in response to influenza infection and have reduced recall responses and protection against re-infection. ICOSL^−/−^ or ICOS^−/−^ and wild-type Balb/c mice were infected i.n. with either the PR8 of influenza then rested (Timeline shown in A). Lung, draining lymph node, and spleen cells were enriched for HA-specific cells and stained for CD4, HA-tetramer, and negatively stained for CD8, CD11c, and CD19 (in one fluorochrome). Data represents the total number of HA-specific CD4 T cells in all organs. A. Diagram of infection, resting time, and rechallenge time. B. Weight loss after primary infection. C. Sample plots of HA-tetramer staining on tetramer-enriched cells and CD44/CD62L staining on HA-tetramer^+^ CD4 T cells from day 39 post-infection (quantified in D). D. (*left*) Total HA-tetramer-specific CD4+ T cells at 39 days after PR8 infection, (*middle*) CD44^+^CD62L^+^ HA-tetramer^+^ CD4^+^ central memory cells, and (*right*) CD44+CD62L- HA-tetramer+ CD4+ effector memory cells. E. Total HA-tetramer-specific CD4 T cells in wild-type and ICOSL^−/−^ mice infected with PR8 then challenged 85 days later with re-infection with VSV-HA. F. Timeline for Hkx31 infection and PR8 challenge. G. Weight loss in ICOS^−/−^ and wild-type mice after infection with Hkx31 followed 60 days later by challenge with PR8 virus.

To determine the number of memory cells that developed in the presence or absence of ICOS costimulation, wild-type and ICOS^−/−^ mice were infected then rested (Figure 5A). As previously seen by Humphreys et al. [30], there was no difference in the weight loss after primary influenza infection (Figure 5B) nor was there any defect in the number of HA-tetramer^+^ CD4 T cells at day 10 post-infection (data not shown) in ICOS^−/−^ as compared to wild-type mice. However, by 39 days post-infection, ICOS^−/−^ mice had significantly fewer EM CD4^+^HA-tetramer^+^ cells than wild-type mice (Figure 5D, representative plots in Figure 5C). Interestingly, the total number of memory cells was not different, because ICOS^−/−^ mice also had significantly more CM CD4^+^HA-tetramer^+^ cells than wild-type mice (Figure 5D). These results demonstrate that ICOS costimulation enhances the development of antigen-specific EM CD4 T cells and potentially reduces the development of central memory cells after infection with influenza.

To determine the responsiveness of the wild-type and ICOS^−/−^ memory CD4 T cells, we re-challenged PR8-infected wild-type and ICOSL^−/−^ mice with a strain of VSV expressing the influenza hemagglutinin molecule, VSV-HA. We found significantly fewer responding HA^+^CD4^+^ cells in ICOSL^−/−^ mice than wild-type mice (Figure 5E). After challenge with VSV-HA, virtually all HA^+^ cells were CD62L^low^, suggesting that this population was highly activated by viral infection (data not shown). Interestingly, this was true in the ICOSL^−/−^ mice as well as wild-type mice, suggesting that although ICOS-deficient memory cells are largely CD62L^high^, these cells are capable of down-regulating CD62L when repeatedly stimulated.

To determine whether the reduced population of EM CD4 T cells in ICOS^−/−^ mice was associated with reduced protection to re-infection, we infected Balb/c and ICOS^−/−^ mice with the influenza virus Hkx31, rested the mice for over 30 days, then challenged with the more lethal strain of influenza, PR8 (Figure 5F). PR8 and HKx31 have previously been found to share eight internal proteins which allow for T-mediated protection to PR8 after Hkx31 infection but to have different coat proteins (hemagglutinin & neuraminidase), preventing humoral protection [31], [32]. After challenge with PR8 virus, wild-type mice lost little weight, but ICOS^−/−^ mice lost nearly as much weight after secondary infection as they had during primary infection with PR8 (Figure 5G and 5B). These findings suggest that ICOS^−/−^ mice had dramatically reduced T-mediated protection to re-infection compared to wild-type mice, associated with the reduced effector memory CD4 T cell population in ICOS^−/−^ mice.

## Discussion

In this study we demonstrate that ICOS costimulation regulates the survival of EM but not CM CD4 T cells. ICOS-deficient mice had significantly fewer EM phenotype CD4 T cells, a defect that became progressively greater with age. Interestingly, ICOS-deficient EM CD4 T cells had no defect in homeostatic proliferation, nor was there a defect in the early development of EM CD4 T cells from activated cells. In contrast, there was significantly reduced survival of ICOS-deficient EM CD4 T cells compared to wild-type EM CD4 T cells. This defect was not observed in ICOS-deficient central memory cells nor did we find any defect in EM or CM CD8 T cells. Furthermore, the defect in effector memory CD4 T cells after infection with influenza was associated with significantly more weight-loss after challenge with a heterosubtypic influenza strain. Collectively these results indicate that ICOS costimulation regulates the long-term survival of EM CD4 T cells that produce cytokines and mediate protection against re-infection.

Our findings may resolve some of the controversy surrounding the role of ICOS in the maintenance of memory CD4 T cells. Although a study by Burmeister et al. found that ICOSL^−/−^ mice have fewer EM phenotype CD4 T cells [33], a second study by Mahajan et al. found no defect in the polyclonal memory CD4 T cell population in ICOS^−/−^ mice [34]. Our findings now demonstrate that ICOS costimulation enhances the survival of EM phenotype CD4 T cells and antigen-specific EM CD4 T cells, but the defect in EM CD4 T cells may be camouflaged by compensatory increases or equivalent numbers of central memory CD4 T cells. Specifically, we found that ICOS^−/−^ and wild-type mice had similar numbers of total CD4 memory cells, akin to the study by Mahajan et al. [34], but closer investigation revealed that ICOS^−/−^ mice had more CM and fewer EM CD4 T cells (Figure 5D). ICOS costimulation might regulate EM CD4 T cell survival by either enhancing the development of EM CD4 T cells that are capable of long-term survival or by enhancing the survival directly, perhaps through continuous anti-apoptotic signaling. In support of the latter possibility, previous studies have shown that ICOS costimulation can enhance the expression of anti-apoptotic molecules in CD4 T cells [35], [36]. Our study therefore supports a role for ICOS costimulation in the maintenance of the EM CD4 T cell population, even when there is no effect of ICOS on the total number of memory cells.

A cohort of individuals with chronic variable immunodeficiency (CVID) have been found to have a genomic deletion resulting in homozygous loss of ICOS [37]. CVID is characterized by reduced antibody titers in the blood, especially class-switched antibodies and increased susceptibility to infection. ICOS costimulation has previously been shown to play an important role in T-dependent B cell responses [38], but this may not be the only way in which ICOS-deficiency impairs immune function in ICOS-deficient CVID patients. Takahashi et al. have previously demonstrated that ICOS-deficient CVID patients did have fewer memory T cells, and the defect was most profound in CD62L^low^ memory CD4 T cells [39]. In this study, we demonstrate that ICOS-deficient EM CD4 T cells have defective survival, and in ICOS-deficient patients, CVID is not usually diagnosed until adulthood [37], a point at which the population of naïve CD4 cells is declining and the body begins to depend more on memory cells [40]. Specifically, effector memory CD4 T cells may play important roles in protection against infection [41], [42], [43], [44]. Our data suggest that individuals with ICOS deficiency may have progressively worsening defects in their EM CD4 T cell mediated protection, perhaps leading to progressively worse immunodeficiency and recurring infections.

Due to its effects in reducing T-dependent B cell responses and T_h_2 responses, ICOS costimulation is under consideration as a potential target for clinical applications such as treatment of asthma and cancer [45], [46]. Our study demonstrates that EM CD4 T cells depend on ICOS costimulation for long-term survival, suggesting that ICOS-blockade might reduce the survival of EM CD4 T cell populations and enhancing ICOS costimulation might augment the survival of these populations. Understanding how ICOS regulates the development and the survival of EM CD4 cells may enhance novel vaccination strategies aimed at inducing protective EM CD4 T cells [41], [44] and regulatory strategies to control CD4 responses in diseases such as atopic asthma that are exacerbated by EM CD4 T cells [47], [48], [49], [50], [51], [52].

## Materials and Methods

### Ethics Statement

All animal studies were done in concordance with principles set forth by the Animal Welfare Act and the National Institutes of Health guidelines for the care and use of animals in biomedical research and were reviewed and approved by the University of Chicago Institutional Animal Care and Use Committee.

### Mice

B6 mice were purchased from Charles Rivers Laboratories (Boston, MA). ICOS^−/−^ (B6.ICOS^−/−^) mice were a generous gift of Dr. Flavell and have been previously described [53]. Balb/c ICOS^−/−^ and B6.ICOSL^−/−^ mice were a gift from Dr. Andrew Welcher (Amgen, Thousand Oaks, CA). DO11.10 and CBy.PL(B6)-Thy1a/ScrJ (Balb/c Thy1.1) mice were purchased from the Jackson Laboratory (Bar Harbor, ME). Balb/c ICOS^−/−^ mice were crossed to DO11.10 mice to generate DO11.10^+^ ICOS^−/−^ mice. Animals were housed in a specific pathogen-free facility maintained by the University of Chicago Animal Resources Center (Chicago, IL).

### DO11.10 adoptive transfer model

DO11.10 and ICOS^−/−^ DO11.10 mice were sacrificed, and spleen and lymph node cells were enriched for T cells by passage over a nylon wool column [54]. T-enriched cells were further enriched for CD62L^+^ naïve cells by MACS separation with anti-CD62L beads (Miltenyi Biotec, Auburn, CA). The CD62L^+^ enriched T cells were then stained with CFSE to track cell division [55]. A sample of enriched cells was stained for CD4, KJ1-26, and CD62L and run on the LSR II flow cytometer to identify the percentage of transgenic cells.

One million CD4^+^KJ1-26^+^ cells were transferred to naïve hosts by i.v. injection in 100 microliters of DMEM media. One day later, the hosts were then immunized intraperitoneally with 200 micrograms of ovalbumin peptide (ovalbumin 323–339, Protein-Peptide Core Facility, University of Chicago, Chicago, IL) and 2500 inactivated *Schistosoma mansonii* eggs (Joel Weinstock, Tufts University–New England Medical Center, Boston, MA), which were inactivated as previously described [56], [57], and serve as a T_h_2 adjuvant [58], [59], [60], [61], [62].

At various time-points after immunization, the hosts were sacrificed, and splenocytes and lymph node cells were collected. Adoptively transferred cells were enriched by MACS sorting of splenocytes and lymph node cells stained with biotinylated KJ1-26 antibody and anti-biotin beads as described by Hataye et al. [63]. KJ1-26-enriched and KJ1-26-depleted cells were counted, stained and analyzed by flow cytometry.

### Influenza Infection

A/PR8/34 (PR8, H1N1) and A/Hkx31 (HKx31, H3N2) were gifts from Dr. Stephen M. Tompkins (University of Georgia, Athens, GA). VSV-HA, expressing the PR8 hemagglutinin protein [64] was a gift from Dr. Elizabeth A. Ramsburg (Duke Univesity Human Vaccine Institute, Durham, NC). Wild-type and ICOS^−/−^ Balb/c mice were infected intranasally with 500 plaque forming units (PFU) of the PR8 strain of influenza. On the specified days after infection, the mice were either sacrificed in order to identify the number of antigen-specific cells or re-infected intranasally with 10,000 PFU of VSV-HA. When mice were sacrificed, broncheoalveolar lavage (BAL) was performed and the spleens, lung-draining mediastinal lymph nodes, and lungs were taken and processed.

To infect and challenge with strains of influenza, wild-type and Balb/c ICOS^−/−^ mice were first infected with 5000 PFU of the Hkx31 strain of influenza. On the specified day after infection, mice were challenged with 5000 PFU of the PR8 strain of influenza. Weight loss was monitored daily after challenge and a cohort of mice were sacrificed five days after challenge to take viral titers.

### Lung Processing

Lungs were perfused with 10 mL PBS with 50 units/mL heparin, injected through the right ventricle of the heart. Then the lung was surgically excised and cut into small pieces. The pieces were incubated in a shaker at 37°C for one hour in DMEM media with 30 U/mL Collagenase IV. Any remaining pieces of tissue were removed by straining the solution through a nytex filter, and the cells in suspension were centrifuged and resuspended in DMEM + 5% FCS.

### Hemagglutinin Tetramer Staining and Enrichment

The hemagglutinin tetramer consists of a PE-labeled streptavidin core binding four biotinylated I-A^d^-HA (143–155) monomer subunits. Tetramers were kindly provided by the NIH Tetramer Core Facility at Emory University (Atlanta, GA). Single cell suspensions were stained with PE-labeled hemagglutinin tetramer by incubating cells with 20 µg/mL of tetramer, diluted in 2.4G2 with 2% mouse and 2% rat serum, for 3 hours at 4°C. Spleens were labeled in 100 microliters of tetramer solution; lymph nodes, lungs, and BAL cells were each labeled in 50 microliters of tetramer solution. After labeling, the cells were washed in MACS buffer then incubated at 4°C for 20 minutes with anti-PE magnetic beads. Finally, bead-labeled cells were washed in MACS buffer then run over magnetic columns to enrich for tetramer-positive cells. All of the tetramer-enriched cells and a sample portion of the tetramer-depleted cells were stained with antibodies and run on a flow cytometer.

### 5-bromo-2-deoxyuridine (BrdU) treatment and analysis

Mice were injected in their intraperitoneal cavity (IP) with 2 mg of BrdU (Sigma-Aldrich, St. Louis, MO) in 200 µL of PBS on a daily basis for 24 days. After 24 days, BrdU treatment was ceased. BrdU uptake was analyzed during and after BrdU treatment on a weekly basis by staining peripheral blood lymphocytes (PBL) isolated from approximately 150 µL of mouse blood. The blood was separated over a Histopaque 1083 (Sigma-Aldrich, St. Louis, MO) gradient, saving the interface cells. The separated viable mononuclear cells were stained for CD4 cell markers first (as described in Flow Cytometry) then for BrdU, using the protocol and reagents from the FITC BrdU Flow Cytometry Kit (BD Biosciences, San Jose, CA). Identification of BrdU positive cells is based on BrdU staining of treated mice versus an untreated control mouse (to identify background staining).

### ELISPOT analysis

Plates were pre-incubated with unlabeled coating antibody for IL-4 (BD Bioscences, San Jose, CA) according to the manufacturer's protocol. For the cell stimulation, KJ-126-enriched cells were incubated on the coated plates for 48 hours with OVA peptide (1 µg/mL) or no antigen and 1.0×10^6^ splenocytes were added as antigen-presenting cells. After cell stimulation, the plates were then sequentially treated with the biotinylated secondary antibody, streptavidin-HRP and AEC (3-Amino-9-ethyl-carbazole) solution according to the manufacturer's protocol. The number of spots was calculated by the ImmunoSpot plate reader (Cellular Technology LTD., Shaker Heights, OH). The number of spots per million cells was calculated based on cellular input and was then multiplied by the total number of cells to find the total number of IL-4 producing cells.

### Flow cytometry

Prior to staining with antibodies, cells were washed once and blocked and nonspecific antibody binding was blocked with anti-FcγR (2.4G2) antibodies. Antibodies were purchased from BD Biosciences (San Jose, CA), eBioscience (San Diego, CA), and Biolegend (San Diego, CA), and included: KJ1-26 (for DO11.10 TCR idiotype), anti-Vα2 (clone B20.1), anti-Vβ5 (MR9-4), anti-CD44 (PGP-1), anti-CD62L (MEL-14), anti-CD4 (RM4-5), anti-CD3 (2C11), anti-ICOS (C398.4A & 7E.17G9), anti-CD69 (H1.2F3), anti-CCR7 (4B12) [Note: CCR7 required staining at 37°C], anti-CD45.1 (Ly5.1, clone A20), and anti-CD45.2 (Ly5.2, clone 104). After primary staining, the cells were washed three times then secondary antibodies (streptavidin-PE, streptavidin-APC, or streptavidin-APC-Cy7) were added and cells were incubated for 30 minutes at 4°C. Then cells were washed three more times and then run on a LSR II flow cytometer (BD Biosciences San Jose, CA) and analyzed using FlowJo software (TreeStar, Ashland OR).

### Data analysis and statistics

Microsoft Excel was utilized to calculate fractions and numbers. Data was then further analyzed and presented using Prizm 5.0 software (GraphPad Software, San Diego, CA). A student's t-test was utilized to determine whether two populations were significantly different. Two-way ANOVA was utilized to determine if one population changed over time differently than another population. If p≤0.05, results were considered significant. *p≤0.05; ** p≤0.01; ***p≤0.001.
